# Tetra­aqua­bis­[5-(pyridin-4-yl)tetra­zolido *N* ^5^-oxide-κ*N* ^2^]manganese(II)

**DOI:** 10.1107/S1600536812032618

**Published:** 2012-07-28

**Authors:** Xiang Jing, Ya Luo

**Affiliations:** a College of Chemistry and Environmental Engineering, Yangtze University, Jingzhou 434020, Hubei, People’s Republic of China

## Abstract

The title compound, [Mn(C_6_H_4_N_5_O)_2_(H_2_O)_4_], is isotypic with its Zn, Ni and Cd analogues reported recently. In the crystal, the Mn^II^ cations are coordinated by four O atoms from four aqua ligands and two N atoms from two 5-(pyridin-4-yl)tetra­zolide N^5^-oxide ligands in a distorted octa­hedral coordination environment. The asymmetric unit consists of one Mn^II^ cation located on a crystallographic twofold axis, and two crystallographically independent water mol­ecules and one *N*-donor ligand in general positions. The discrete complex mol­ecules are arranged in alternating rows parallel to [100] and are linked by O—H⋯N and O—H⋯O hydrogen bonds into a three-dimensional network.

## Related literature

For related structures, see: Yang *et al.* (2009 ▶); Yu *et al.* (2004*a* ▶,*b* ▶). For the coordination properties of tetra­zolate ligands, see: Aromí *et al.* (2011 ▶).
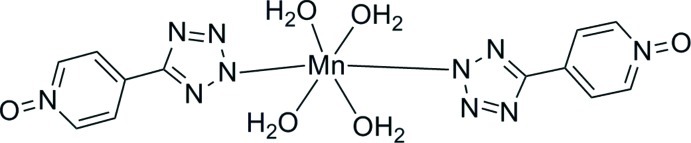


## Experimental

### 

#### Crystal data

[Mn(C_6_H_4_N_5_O)_2_(H_2_O)_4_]
*M* *_r_* = 451.29
Monoclinic, 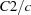

*a* = 21.828 (2) Å
*b* = 7.0620 (9) Å
*c* = 11.3229 (13) Å
β = 96.515 (10)°
*V* = 1734.1 (3) Å^3^
*Z* = 4
Mo *K*α radiation
μ = 0.82 mm^−1^
*T* = 293 K
0.32 × 0.25 × 0.20 mm

#### Data collection

Bruker APEX area-dectector diffractometer
Absorption correction: multi-scan (*SADABS*; Sheldrick, 1996 ▶) *T*_min_ = 0.782, *T*_max_ = 0.849
5196 measured reflections
1530 independent reflections
1149 reflections with *I* > 2σ(*I*)
*R*_int_ = 0.049

#### Refinement

*R*[*F* ^2^ > 2σ(*F* ^2^)] = 0.037
*wR*(*F* ^2^) = 0.080
*S* = 1.05
1530 reflections
145 parameters
H atoms treated by a mixture of independent and constrained refinement
Δρ_max_ = 0.21 e Å^−3^
Δρ_min_ = −0.25 e Å^−3^

### 

Data collection: *APEX2* (Bruker, 2004 ▶); cell refinement: *SAINT* (Bruker, 2004 ▶); data reduction: *SAINT*; program(s) used to solve structure: *SHELXS97* (Sheldrick, 2008 ▶); program(s) used to refine structure: *SHELXL97* (Sheldrick, 2008 ▶); molecular graphics: *SHELXTL* (Sheldrick, 2008 ▶); software used to prepare material for publication: *SHELXL97*.

## Supplementary Material

Crystal structure: contains datablock(s) I, global. DOI: 10.1107/S1600536812032618/nc2281sup1.cif

Structure factors: contains datablock(s) I. DOI: 10.1107/S1600536812032618/nc2281Isup2.hkl

Supplementary material file. DOI: 10.1107/S1600536812032618/nc2281Isup3.cdx

Additional supplementary materials:  crystallographic information; 3D view; checkCIF report

## Figures and Tables

**Table 1:** Hydrogen-bond geometry (Å, °)

*D*—H⋯*A*	*D*—H	H⋯*A*	*D*⋯*A*	*D*—H⋯*A*
O2—H9⋯O3^i^	0.87 (3)	1.80 (3)	2.658 (3)	170 (3)
O1—H11⋯O3^ii^	0.80 (3)	1.98 (3)	2.770 (3)	173 (3)
O2—H10⋯O3^iii^	0.87 (3)	1.88 (3)	2.751 (3)	172 (3)
O1—H12⋯N3^iv^	0.81 (3)	2.05 (3)	2.861 (3)	171 (3)

Symmetry codes: (i) 
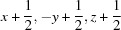
; (ii) 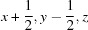; (iii) 
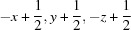
; (iv) 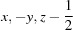.

**Fig. 1.**
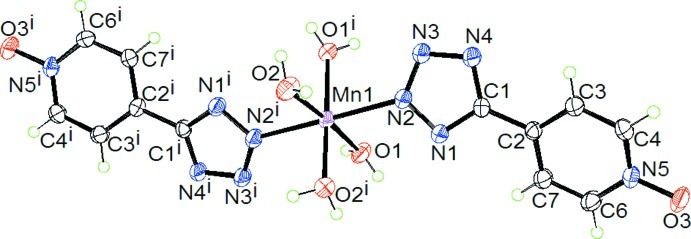
ORTEP drawing of the title compound with labeling. Displacement ellipsoids are drawn at 30% probability level and H atoms are drawn as spheres of arbitrary radius. [Symmetry code: -x + 1, y, -z + 1/2]

**Fig. 2.**
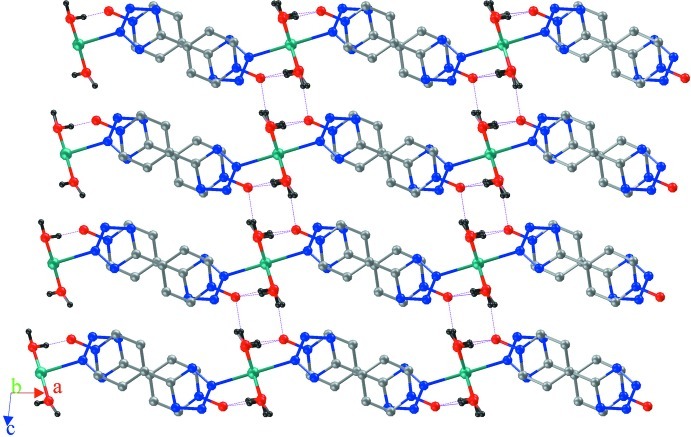
The packing diagram of the title compound with intermolecular H bonding along ac plane shown as dashed lines.

## supplementary crystallographic information

### Comment

Ligands based on tetrazolates have attracted wide attention because of their versatile coordination modes and therefore, a large number of metal complexes containing these types of ligands are reported in literature (Aromí *et al.*, 2011). Several of them have been prepared *via in situ* synthesis of nitriles and azides. In view of this we have reacted 4-cyanopyridine 1-oxide with manganese chloride and sodium azide, which results in the formation of crystals of the title compound that is isotypic to its Zn, Ni and Cd analogs (Yu *et al.*, 2004*a*,*b*; Yang *et al.*, 2009).

The coordination geometry of the Mn atom is a slightly distorted octahedron formed by the coordination of four water molecules and two tetrazolate ligands (Fig. 1). The oxygen atoms from the four water molecules form a square planar arrangement around the Mn center and the tetrazolate ligands coordinate *via* the N atom to the Mn cations. The discrete complex molecules are arranged in alternating rows parallel to [100] and are linked by O—H···N and O—H···O hydrogen bonds into a three-dimensional network. (Fig. 2 and Table 1)

### Experimental

The mixture of 4-cyanopyridine 1-oxide (0.42 mmol, 50.3 mg), MnCl_2_.6H_2_O (0.50 mmol, 116.5 mg) and NaN_3_ (0.70 mmol, 45.6 mg) in 15 ml EtOH and H_2_O (v/v = 2:1) were heated in a 25 ml bomb at 393 K for 3 d, then cooled to room temperature at a rate of 6 K h^-1^. Colorless block-shaped crystals suitable for X-ray analysis were obtained in a yield of 40% based on the ligand 4-cyanopyridine 1-oxide. The product was washed with EtOH and H_2_O, and then air-dried at ambient temperature. Elemental analysis for C_12_H_16_N_10_O_6_Mn found: C 31.75, H 3.52, N 30.97; calculated: C 31.94, H 3.57, N 31.04. Selected IR (KBr, cm^-1^): 3118 (*w*), 3058(*w*), 1532 (*m*), 1462 (*m*), 1439 (*m*), 1215 (*s*), 1193 (*s*), 856 (*s*).

### Refinement

Hydrogen atoms were placed in calculated positions (C—H 0.93 Å; U = 1.2*U*_eq_C), and were included in the refinement in the riding model approximation. The hydrogen atoms of aqua ligands were located and refined.

### Crystal data

**Table:**

[Mn(C_6_H_4_N_5_O)_2_(H_2_O)_4_]	*F*(000) = 924
*M**_r_* = 451.29	*D*_x_ = 1.729 Mg m^−^^3^
Monoclinic, *C*2/*c*	Mo *K*α radiation, λ = 0.71073 Å
Hall symbol: -C 2yc	Cell parameters from 65 reflections
*a* = 21.828 (2) Å	θ = 2.2–26.0°
*b* = 7.0620 (9) Å	µ = 0.82 mm^−^^1^
*c* = 11.3229 (13) Å	*T* = 293 K
β = 96.515 (10)°	Block, colourless
*V* = 1734.1 (3) Å^3^	0.32 × 0.25 × 0.20 mm
*Z* = 4	

### Data collection

**Table:**

Bruker APEX area-dectector diffractometer	1530 independent reflections
Radiation source: fine-focus sealed tube	1149 reflections with *I* > 2σ(*I*)
Graphite monochromator	*R*_int_ = 0.049
φ and ω scans	θ_max_ = 25.0°, θ_min_ = 3.0°
Absorption correction: multi-scan (*SADABS*; Sheldrick, 1996)	*h* = −24→25
*T*_min_ = 0.782, *T*_max_ = 0.849	*k* = −8→7
5196 measured reflections	*l* = −13→13

### Refinement

**Table:**

Refinement on *F*^2^	Primary atom site location: structure-invariant direct methods
Least-squares matrix: full	Secondary atom site location: difference Fourier map
*R*[*F*^2^ > 2σ(*F*^2^)] = 0.037	Hydrogen site location: inferred from neighbouring sites
*wR*(*F*^2^) = 0.080	H atoms treated by a mixture of independent and constrained refinement
*S* = 1.05	*w* = 1/[σ^2^(*F*_o_^2^) + (0.0291*P*)^2^] where *P* = (*F*_o_^2^ + 2*F*_c_^2^)/3
1530 reflections	(Δ/σ)_max_ < 0.001
145 parameters	Δρ_max_ = 0.21 e Å^−^^3^
0 restraints	Δρ_min_ = −0.25 e Å^−^^3^

### Special details

**Table:**

Geometry. All e.s.d.'s (except the e.s.d. in the dihedral angle between two l.s. planes) are estimated using the full covariance matrix. The cell e.s.d.'s are taken into account individually in the estimation of e.s.d.'s in distances, angles and torsion angles; correlations between e.s.d.'s in cell parameters are only used when they are defined by crystal symmetry. An approximate (isotropic) treatment of cell e.s.d.'s is used for estimating e.s.d.'s involving l.s. planes.
Refinement. Refinement of *F*^2^ against ALL reflections. The weighted *R*-factor *wR* and goodness of fit *S* are based on *F*^2^, conventional *R*-factors *R* are based on *F*, with *F* set to zero for negative *F*^2^. The threshold expression of *F*^2^ > σ(*F*^2^) is used only for calculating *R*-factors(gt) *etc*. and is not relevant to the choice of reflections for refinement. *R*-factors based on *F*^2^ are statistically about twice as large as those based on *F*, and *R*-factors based on ALL data will be even larger.

### Fractional atomic coordinates and isotropic or equivalent isotropic displacement parameters (Å^2^)

**Table:**

	*x*	*y*	*z*	*U*_iso_*/*U*_eq_	
Mn1	0.5000	0.16002 (8)	0.2500	0.0261 (2)	
N1	0.35497 (9)	0.1653 (3)	0.2280 (2)	0.0300 (6)	
N2	0.40483 (10)	0.1527 (3)	0.3078 (2)	0.0296 (5)	
N3	0.38821 (10)	0.1262 (3)	0.4150 (2)	0.0328 (6)	
N4	0.32672 (10)	0.1218 (3)	0.4086 (2)	0.0320 (6)	
O1	0.47398 (9)	−0.0641 (3)	0.12179 (19)	0.0344 (5)	
O2	0.52626 (10)	0.3978 (3)	0.3678 (2)	0.0380 (6)	
O3	0.06246 (8)	0.1620 (3)	0.09684 (18)	0.0383 (5)	
C1	0.30785 (11)	0.1464 (4)	0.2926 (2)	0.0236 (6)	
C2	0.24297 (11)	0.1479 (3)	0.2420 (2)	0.0229 (6)	
C3	0.19542 (12)	0.1790 (3)	0.3111 (3)	0.0296 (6)	
H3	0.2044	0.1985	0.3924	0.036*	
C4	0.13523 (12)	0.1815 (4)	0.2608 (3)	0.0311 (7)	
H4	0.1037	0.2006	0.3084	0.037*	
N5	0.12168 (9)	0.1565 (3)	0.1437 (2)	0.0282 (5)	
C6	0.16617 (12)	0.1269 (4)	0.0737 (3)	0.0327 (7)	
H6	0.1558	0.1112	−0.0077	0.039*	
C7	0.22684 (12)	0.1197 (4)	0.1205 (3)	0.0304 (7)	
H7	0.2573	0.0959	0.0713	0.036*	
H9	0.5404 (16)	0.367 (4)	0.440 (3)	0.058 (5)*	
H11	0.5008 (16)	−0.137 (4)	0.111 (3)	0.058 (5)*	
H10	0.4956 (15)	0.475 (4)	0.376 (3)	0.058 (5)*	
H12	0.4499 (16)	−0.069 (4)	0.061 (3)	0.058 (5)*	

### Atomic displacement parameters (Å^2^)

**Table:**

	*U*^11^	*U*^22^	*U*^33^	*U*^12^	*U*^13^	*U*^23^
Mn1	0.0187 (3)	0.0362 (4)	0.0234 (4)	0.000	0.0021 (2)	0.000
N1	0.0206 (13)	0.0446 (14)	0.0245 (13)	−0.0004 (10)	0.0011 (10)	0.0032 (12)
N2	0.0205 (12)	0.0402 (13)	0.0275 (14)	0.0003 (10)	0.0003 (10)	0.0011 (12)
N3	0.0210 (13)	0.0499 (15)	0.0274 (14)	0.0011 (10)	0.0023 (11)	0.0025 (12)
N4	0.0210 (13)	0.0494 (15)	0.0258 (14)	0.0031 (10)	0.0038 (10)	0.0009 (12)
O1	0.0257 (13)	0.0483 (13)	0.0278 (12)	0.0033 (9)	−0.0032 (9)	−0.0070 (11)
O2	0.0309 (13)	0.0486 (14)	0.0334 (13)	0.0065 (9)	−0.0014 (10)	−0.0086 (11)
O3	0.0202 (10)	0.0496 (12)	0.0429 (13)	−0.0007 (9)	−0.0065 (9)	−0.0007 (11)
C1	0.0189 (14)	0.0254 (14)	0.0267 (16)	−0.0009 (11)	0.0033 (12)	−0.0010 (13)
C2	0.0205 (14)	0.0222 (14)	0.0257 (15)	−0.0017 (11)	0.0011 (12)	0.0036 (14)
C3	0.0236 (15)	0.0409 (16)	0.0245 (15)	−0.0020 (12)	0.0033 (12)	−0.0017 (14)
C4	0.0232 (15)	0.0427 (18)	0.0286 (16)	0.0007 (12)	0.0086 (12)	−0.0010 (15)
N5	0.0188 (12)	0.0314 (12)	0.0339 (14)	−0.0004 (10)	0.0011 (10)	0.0002 (12)
C6	0.0275 (16)	0.0464 (18)	0.0235 (16)	0.0020 (13)	−0.0005 (13)	−0.0061 (14)
C7	0.0229 (15)	0.0418 (17)	0.0268 (16)	0.0040 (12)	0.0045 (13)	−0.0013 (14)

### Geometric parameters (Å, º)

**Table:**

Mn1—O1	2.179 (2)	O2—H10	0.87 (3)
Mn1—O1^i^	2.179 (2)	O3—N5	1.341 (3)
Mn1—O2	2.180 (2)	C1—C2	1.467 (3)
Mn1—O2^i^	2.180 (2)	C2—C3	1.387 (4)
Mn1—N2^i^	2.248 (2)	C2—C7	1.394 (4)
Mn1—N2	2.248 (2)	C3—C4	1.371 (4)
N1—C1	1.335 (3)	C3—H3	0.9300
N1—N2	1.336 (3)	C4—N5	1.338 (3)
N2—N3	1.319 (3)	C4—H4	0.9300
N3—N4	1.336 (3)	N5—C6	1.338 (4)
N4—C1	1.342 (3)	C6—C7	1.371 (3)
O1—H11	0.80 (3)	C6—H6	0.9300
O1—H12	0.81 (3)	C7—H7	0.9300
O2—H9	0.87 (3)		
			
O1—Mn1—O1^i^	86.82 (11)	Mn1—O2—H9	115 (2)
O1—Mn1—O2	175.99 (9)	Mn1—O2—H10	113 (2)
O1^i^—Mn1—O2	96.97 (8)	H9—O2—H10	104 (3)
O1—Mn1—O2^i^	96.97 (8)	N1—C1—N4	112.3 (2)
O1^i^—Mn1—O2^i^	175.99 (9)	N1—C1—C2	123.7 (2)
O2—Mn1—O2^i^	79.27 (12)	N4—C1—C2	124.0 (2)
O1—Mn1—N2^i^	88.23 (8)	C3—C2—C7	117.2 (2)
O1^i^—Mn1—N2^i^	89.86 (8)	C3—C2—C1	122.1 (2)
O2—Mn1—N2^i^	90.47 (8)	C7—C2—C1	120.6 (2)
O2^i^—Mn1—N2^i^	91.55 (8)	C4—C3—C2	120.8 (3)
O1—Mn1—N2	89.86 (8)	C4—C3—H3	119.6
O1^i^—Mn1—N2	88.23 (8)	C2—C3—H3	119.6
O2—Mn1—N2	91.55 (8)	N5—C4—C3	120.2 (3)
O2^i^—Mn1—N2	90.47 (8)	N5—C4—H4	119.9
N2^i^—Mn1—N2	177.37 (12)	C3—C4—H4	119.9
C1—N1—N2	104.0 (2)	C4—N5—C6	121.0 (2)
N3—N2—N1	110.08 (19)	C4—N5—O3	118.9 (2)
N3—N2—Mn1	129.10 (16)	C6—N5—O3	120.1 (2)
N1—N2—Mn1	120.71 (17)	N5—C6—C7	120.7 (3)
N2—N3—N4	109.4 (2)	N5—C6—H6	119.6
N3—N4—C1	104.2 (2)	C7—C6—H6	119.6
Mn1—O1—H11	115 (3)	C6—C7—C2	120.1 (3)
Mn1—O1—H12	133 (2)	C6—C7—H7	120.0
H11—O1—H12	105 (4)	C2—C7—H7	120.0
			
C1—N1—N2—N3	0.4 (3)	N3—N4—C1—N1	0.1 (3)
C1—N1—N2—Mn1	176.92 (17)	N3—N4—C1—C2	178.7 (2)
O1—Mn1—N2—N3	123.9 (2)	N1—C1—C2—C3	−162.3 (3)
O1^i^—Mn1—N2—N3	37.1 (2)	N4—C1—C2—C3	19.3 (4)
O2—Mn1—N2—N3	−59.8 (2)	N1—C1—C2—C7	17.0 (4)
O2^i^—Mn1—N2—N3	−139.1 (2)	N4—C1—C2—C7	−161.5 (3)
N2^i^—Mn1—N2—N3	80.5 (2)	C7—C2—C3—C4	0.0 (4)
O1—Mn1—N2—N1	−51.82 (19)	C1—C2—C3—C4	179.3 (2)
O1^i^—Mn1—N2—N1	−138.64 (19)	C2—C3—C4—N5	−1.0 (4)
O2—Mn1—N2—N1	124.43 (19)	C3—C4—N5—C6	0.6 (4)
O2^i^—Mn1—N2—N1	45.15 (19)	C3—C4—N5—O3	−179.1 (2)
N2^i^—Mn1—N2—N1	−95.22 (18)	C4—N5—C6—C7	0.7 (4)
N1—N2—N3—N4	−0.4 (3)	O3—N5—C6—C7	−179.6 (2)
Mn1—N2—N3—N4	−176.50 (17)	N5—C6—C7—C2	−1.6 (4)
N2—N3—N4—C1	0.2 (3)	C3—C2—C7—C6	1.2 (4)
N2—N1—C1—N4	−0.3 (3)	C1—C2—C7—C6	−178.1 (2)
N2—N1—C1—C2	−178.9 (2)		

Symmetry code: (i) −*x*+1, *y*, −*z*+1/2.

### Hydrogen-bond geometry (Å, º)

**Table:**

*D*—H···*A*	*D*—H	H···*A*	*D*···*A*	*D*—H···*A*
O2—H9···O3^ii^	0.87 (3)	1.80 (3)	2.658 (3)	170 (3)
O1—H11···O3^iii^	0.80 (3)	1.98 (3)	2.770 (3)	173 (3)
O2—H10···O3^iv^	0.87 (3)	1.88 (3)	2.751 (3)	172 (3)
O1—H12···N3^v^	0.81 (3)	2.05 (3)	2.861 (3)	171 (3)

Symmetry codes: (ii) *x*+1/2, −*y*+1/2, *z*+1/2; (iii) *x*+1/2, *y*−1/2, *z*; (iv) −*x*+1/2, *y*+1/2, −*z*+1/2; (v) *x*, −*y*, *z*−1/2.

## References

[bb1] Aromí, G. L., Barrios, A., Roubeau, O. & Gamez, P. (2011). *Coord. Chem. Rev.* **255**, 485–546.

[bb2] Bruker (2004). *APEX2* and *SAINT* Bruker AXS Inc., Madison, Wisconsin, USA.

[bb3] Sheldrick, G. M. (1996). *SADABS* University of Göttingen, Germany.

[bb4] Sheldrick, G. M. (2008). *Acta Cryst.* A**64**, 112–122. 10.1107/S0108767307043930 18156677

[bb5] Yang, W. B., Lin, X., Blake, A. J., Wilson, C., Hubberstey, P., Champness, N. R. & Schröder, M. (2009). *CrystEngComm*, **11**, 67–81.

[bb6] Yu, Z.-X., Wang, X.-P. & Feng, Y. (2004*a*). *Acta Cryst.* C**60**, m194–m196. 10.1107/S0108270104005864 15071216

[bb7] Yu, Z. X., Wang, X. P., Feng, Y. Y. & Zhong, X. H. (2004*b*). *Inorg. Chem. Commun.* **7**, 492–494.

